# Prevalence and epidemiology of canine and feline heartworm infection in Taiwan

**DOI:** 10.1186/s13071-017-2435-7

**Published:** 2017-11-09

**Authors:** Ta-Li Lu, Jun-Yue Wong, Ta-Lun Tan, Yong-Wei Hung

**Affiliations:** 1 Taiwan Academy of Veterinary Internal Medicine, Taipei, Taiwan; 2 Cardiospecial Veterinary Hospital, No.34, Sec. 2, Heping E. Rd, Taipei, 106 Taiwan; 3 Cambridge Animal Hospital, Taipei, Taiwan; 4 Manhattan Veterinary Hospital, Taipei, Taiwan

**Keywords:** Heartworm infection, Taiwan, Canine, Feline, Heartworm prevalence, Antigen testing, Antibody testing

## Abstract

**Background:**

Heartworm, *Dirofilaria immitis,* has long been recognized in Taiwanese dogs but feline heartworm infection has been largely overlooked by veterinarians and pet owners. The main goal of this study was to determine the prevalence and epidemiology of canine and feline heartworm infection in Taiwan.

**Methods:**

Household dogs and cats were selected from 103 veterinary hospitals in 13 cities throughout Taiwan. All animals were at least 1 year old, had received no heartworm prevention for more than 1 year, and had lived in the same city for at least 1 year. Client consent was obtained and an owner questionnaire was completed for each patient. Blood samples were collected from each canine patient and tested at each veterinary hospital for microfilariae and for circulating antigen. A positive result on either test was considered to confirm mature heartworm infection. Blood was collected from each feline patient and examined for microfilariae and a feline heartworm antigen/antibody test was performed. Descriptive statistics were used for heartworm prevalence. Multivariate logistic regression analysis was used to determine the relationships between heartworm infection and multiple risk factors.

**Results:**

A total of 2064 household dogs and 616 household cats from 103 veterinary hospitals throughout Taiwan were included in the study. The overall prevalence of canine heartworm disease was 22.8% (471/2064). In heartworm-positive dogs, 63% were both microfilaria positive and antigen positive, 35% were microfilaria negative and antigen positive, and only 2% were microfilaria positive and antigen negative. In the comparison of different life style groups, outdoor dogs (*N* = 797) had significantly higher heartworm prevalence rate than indoor dogs (*N* = 1267; *p* = 0.000). The heartworm prevalence rate in dogs presented with dyspnea and cough was as high as 51%. The overall prevalence of antibody-positive cats was 6.7% (41/616) and the antigen-positive prevalence rate was 3.1% (19/616). In 41 antibody-positive cats, 6 of them were also antigen-positive. In 19 antigen-positive cats, 13 of them were antibody negative. In antibody-positive and antigen-negative cats, half had no clinical signs. In antigen-positive cats, 21% had no clinical signs and only 38% had classic heartworm clinical signs (dyspnea, cough, or gastrointestinal signs).

**Conclusions:**

Our canine study showed that southern and eastern Taiwan have the highest heartworm prevalence. Dogs not receiving preventive and living outdoors or those that have either cough or dyspnea have a high incidence of heartworm infection. We also confirmed that feline heartworm exposure exists in most cities in Taiwan. The diagnosis of feline heartworm infection will remain challenging for clinicians, however, without a consistent relationship between the presence of heartworm infection and clinical signs and the vagaries of microfilaria and antigen/antibody testing.

**Electronic supplementary material:**

The online version of this article (10.1186/s13071-017-2435-7) contains supplementary material, which is available to authorized users.

## Background

Heartworm, *Dirofilaria immitis,* is one of the most common and devastating parasitic disease in dogs. Taiwan, with its location in East Asia and its marine tropical climate, is a perfect environment for heartworm disease. Canine heartworm was first reported in Taiwan in 1935 [1]. There have been many reports of canine heartworm prevalence since then [2–11]. In 2001, Fan et al. investigated 523 pet dogs in Taipei and 141 dogs in mountain aboriginal districts and the heartworm prevalence was 13.8% (72/523) and 12.1% (17/141), respectively [12]. In 2003, a large scale study was published with 837 stray dogs and 1228 pet dogs from 13 cities throughout Taiwan. The overall heartworm prevalence rate in stray dogs and pet dogs was 57% and 26.5%, respectively. The highest rate in pet dogs was found in Nantou (40.7%) and lowest rate was in Hualien (4%) [13]. None of these reports included the percentage of dogs on preventive. No large-scale studies on the prevalence of canine heartworm infection (HWI) in Taiwan have been published since then.

On the other hand, HWI in cats has been largely overlooked by veterinarians and owners. Compared with dogs, the diagnosis of feline HWI is more challenging. The first prevalence study of HWI in cats in Taipei city found only one antigen (Ag)-positive cat among 200 pet cats [14]. Recently, another study focusing on relationship between feline heartworm and lower airway signs revealed an antibody (Ab) seroprevalence of 9.7% in 226 pet cats in Taipei city [15]. There have been no large-scale studies of feline HWI prevalence involving different cities in Taiwan.

Many new heartworm diagnostic methods and preventive medications for both dogs and cats have been introduced in Taiwan in the last decade. We assume that those newer tools may affect the known prevalence and epidemiology of HWI in Taiwan and practitioners need updated information to educate clients and treat and prevent heartworm disease. This study is the first large-scale study of feline heartworm disease prevalence in Taiwan and also the largest study of canine heartworm prevalence in pet dogs in Taiwan to date.

The goal of the study was to determine the current heartworm prevalence in pet dogs and cats in Taiwan and the potential risk factors that might have influence on heartworm prevalence.

## Methods

**Fig. 1:**
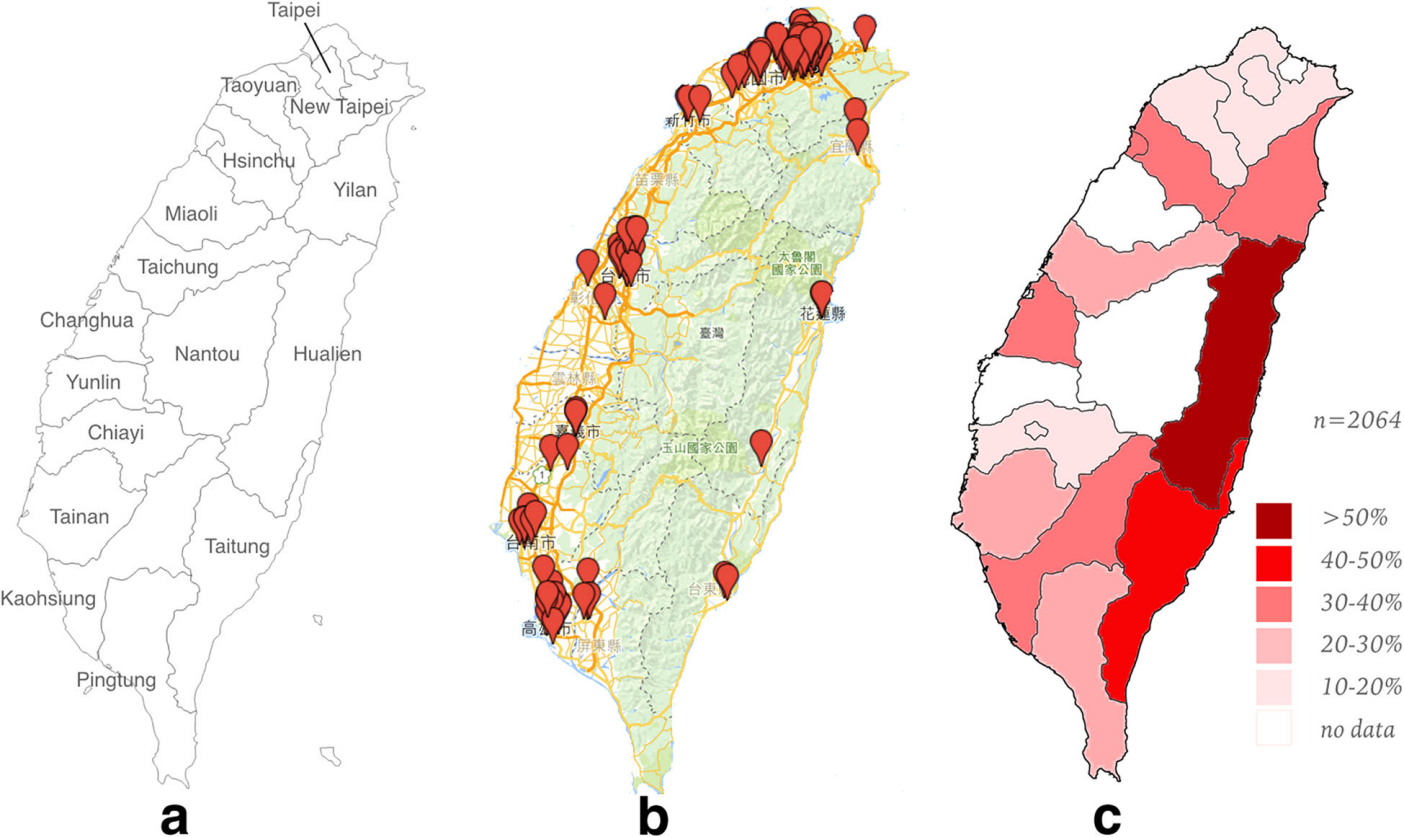
**a** Map of Taiwan. **b** Study sampling locations. **c** Canine heartworm prevalence in Taiwan

Household dogs and cats were selected from 103 veterinary hospitals in 13 cities throughout Taiwan (Fig. 1). The number of hospitals involved in each city was decided based on the populations of pet dogs and cats. The target was evaluation of 30 dogs and 10 cats from each hospital. Dogs and cats included in this study were at least 1 year old, had received no heartworm prevention for more than 1 year, and had lived in the same city for at least 1 year. The study was approved by the ethical committee of Taiwan Academy of Veterinary Internal Medicine. For each patient, client consent was obtained and a questionnaire, including signalment, body weight, lifestyle (100% of the time indoors, 100% of the time outdoors, >50% indoors or >50% outdoors), clinical signs at presentation [no clinical sign, dyspnea, cough, gastrointestinal (GI) sign or other clinical signs] was completed.

All blood samples were collected and tested at each veterinary hospital and the results were sent to the investigators using an online form. For each canine patient, at least 1 mL of blood was collected. The blood in EDTA tube was examined for circulating Ag (0.5 mL) using commercial kit (SNAP® 4Dx® Plus, IDEXX Laboratories, Westbrook, Maine, USA) and for microfilariae (0.5 mL) using a direct blood smear technique by placing 0.5 mL blood sample from EDTA tube on the slide with cover slips over top and examination under the microscope. Either Ag or microfilariae positive was considered to confirm HWI.

For each feline patient, at least 2.5 mL of blood was collected in EDTA tubes and was examined for microfilariae (0.5 mL), also using a direct blood smear technique. Serum was collected from the non-anticoagulant tube and the feline heartworm Ab test was done using a commercial kit (Solo Step® FH, Heska, Loveland, Colorado, USA) and the Ag test was performed using a commercial kit (SNAP® Feline Heartworm, IDEXX Laboratories; Witness® HW, Zoetis, Parsippany, New Jersey, USA).

Descriptive statistics were used to demonstrate heartworm prevalence. Prevalence results are expressed as median (range). Multivariate logistic regression analysis was used to determine the relationships between heartworm prevalence and the presence of multiple risk factors obtained from the questionnaire.

## Results

### Dogs

In the canine study, 2064 pet dogs were included and tested for heartworm disease from April to October 2015. There were 1071 male and 993 female dogs, with a median age of 7 years (range from 1 to 19 years old) and median body weight of 8.3 kg (range from 1 to 60 kg). The pet dogs included 655 mixed-breed dogs, 253 Maltese, 221 Miniature Poodles, 121 Dachshunds, 120 Miniature Schnauzers, 102 Chihuahuas, 57 Golden Retrievers, 45 Beagles, 42 Labrador retrievers, 38 Shih-Tzus, 37 Pomeranians, 28 Spitzs, and 345 dogs representing other breeds.

The overall prevalence rate of canine heartworm disease was 22.8%, ranging from 12.1% to 51.1% in different cities (Table 1). Hualien had highest prevalence rate of 51.1% (44/86), followed by Taitung (46.7%, 14/30) and Yilan (36.36%, 12/33). Taipei had lowest prevalence rate of 12.1% (44/365), followed by New Taipei City (13.6%, 55/406) and Chiayi (13.9%, 5/36). Overall, eastern and southern Taiwan had higher prevalence rates than northern Taiwan (Fig. 1).

**Table 1:** Prevalence of canine heartworm disease in different cities in Taiwan

Area	Sample Number	Heartworm (+) Number	Heartworm (+) %
Hualien	86	44	51.16%
Taitung	30	14	46.67%
Yilan	33	12	36.36%
Hsinchu	44	15	34.09%
Kaohsiung	316	104	32.91%
Changhua	44	14	31.82%
Taichung	280	70	25.00%
Tainan	237	59	24.89%
Pingtung	35	7	20.00%
Taoyuan	152	28	18.42%
Chiayi	36	5	13.89%
New Taipei	406	55	13.55%
Taipei	365	44	12.05%
Total	2064	471	22.82%

**Fig. 2:**
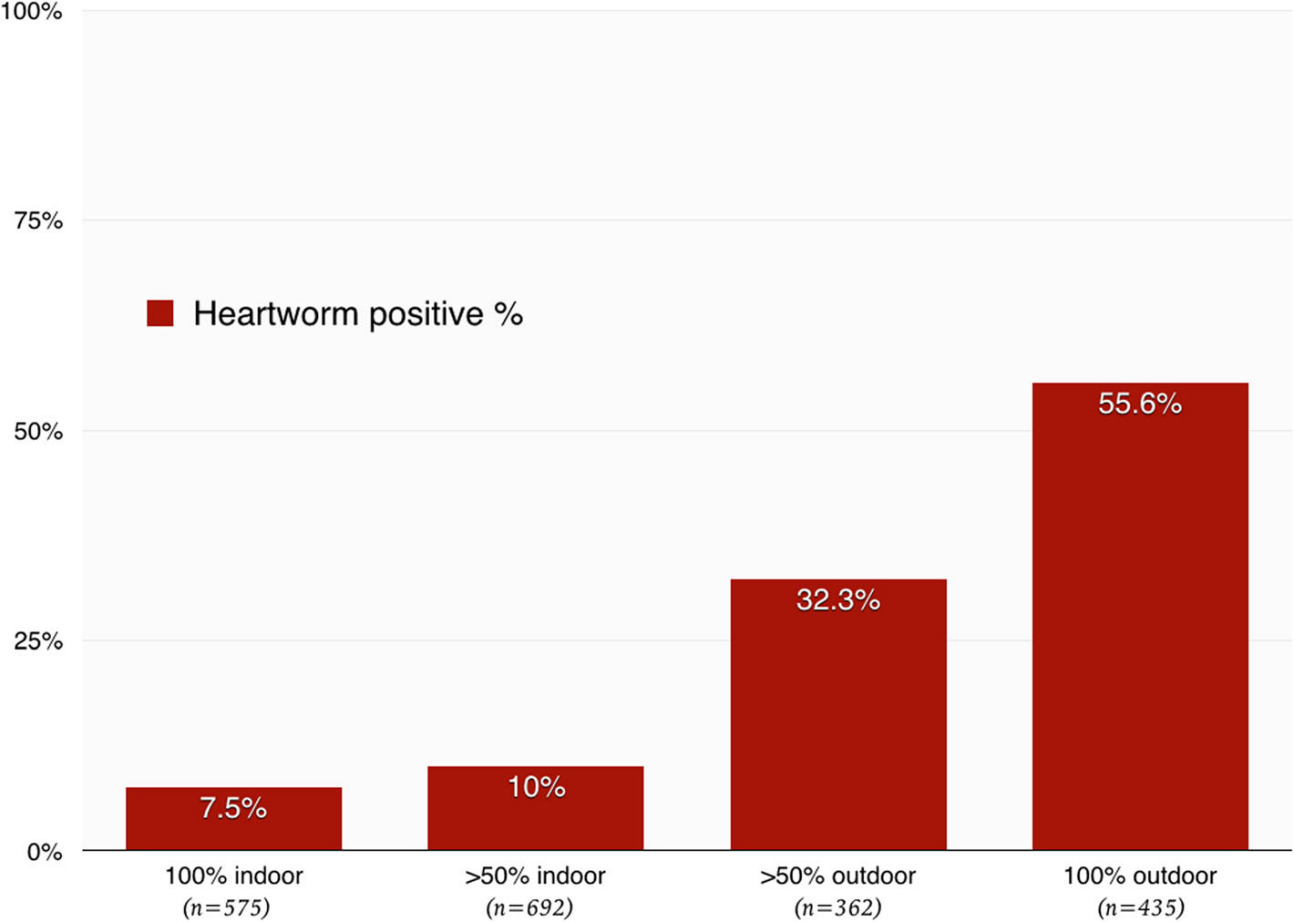
Prevalence of canine heartworm disease in different lifestyle groups (indoor vs outdoor)

Among all dogs, 28% (575/2064) lived 100% indoors, 33% (692/2064) lived >50% indoors, 17% (362/2064) lived >50% outdoors, and 21% (435/2064) lived 100% outdoors. Comparing the different lifestyle groups, the 100% outdoor group had the highest prevalence rate of 55.6%, followed by 32.3% of the >50% outdoor group, 10% of the >50% indoor group, and 7.5% of the 100% indoor group (Fig. 2).

The prevalence difference between each lifestyle group was also noted in each city. For example, in Taipei, which had lowest prevalence rate in Taiwan, canine HWIs were still frequently observed in the 100% outdoor group with prevalence rate of 53% (16/30), followed by 21% (15/70) of the >50% outdoor group, 7% (10/143) of the >50% indoor group, and 2% (3/122) of the 100% indoor group. This high frequency of indoor housing resulted in <13% of dogs being Ag-positive in Taipei. In Hualien, the 100% outdoor group had highest prevalence rate of 70% (35/50), followed by 40% (2/5) of the >50% outdoor group, 31% (4/13) of the >50% indoor group, and 17% (3/18) of the 100% indoor group. The high frequency of outdoor housing resulted in >50% of dogs being Ag-positive in Hualien. In heartworm-positive dogs, 63% were both microfilaria positive and Ag positive, 35% were microfilaria negative and Ag positive, and only 2% were microfilaria positive and Ag negative.

Of all the dogs included in this study, 55% (1125/2064) presented without clinical signs, 14% (282/2064) had cough, 6% (120/2064) had dyspnea, 6% (132/2064) had gastrointestinal signs, and 20% (405/2064) had other clinical signs. In heartworm-positive dogs, 34% (159/471) presented without clinical signs, 28% (133/471) had cough, 13% (61/471) had dyspnea, 4% (18/471) had GI signs, and 21% (100/471) had other clinical signs. On the other hand, in dogs presented without clinical signs, 14% (159/1125) were heartworm positive. The heartworm prevalence rate in dogs presented with cough was 47% (133/282) and dogs presented with dyspnea was 51% (61/120).

Multiple logistic regression results for risk factors of canine HWI are shown in Table 2. We found that spending >50% of time outdoors and having cough or dyspnea were independent risk factors for canine HWI.

**Table 2:** Results of multiple logistic regression on risk factors for canine heartworm disease

		Regression Coefficient (B)	Standard Error (SE)	*P* value	Odds Ratio (OR)	95% Confidence Interval (CI)
Lifestyle	100% indoor	reference				
	> 50% indoor	0.161	0.209	0.441	1.174	0.780–1.768
	> 50% outdoor	1.700	0.201	0.000*	5.475	3.694–8.115
	100% outdoor	2.591	0.193	0.000*	13.340	9.142–19.464
Dyspnea	No	reference				
	Yes	1.370	0.233	0.000*	3.934	2.493–6.208
GI signs	No	reference				
	Yes	−0.265	0.288	0.358	0.767	0.436–1.350
Cough	No	reference				
	Yes	1.657	0.167	0.000*	5.242	3.779–7.273
Other clinical signs	No	reference				
	Yes	0.415	0.160	0.009*	1.515	1.108–2.071

*Significant

### Cats

In the feline study, 616 pet cats were tested for HWI from April to October 2015. There were 327 male and 289 female cats, with a median age of 6 years (range 1 to 20 years) and median body weight of 4 kg (range 1.5 to 9.5 kg). We included 415 Domestic shorthair, 69 Persian, 56 Chinchilla, 30 American Shorthair, 15 Scottish Fold, 8 British Shorthair, 5 Himalayan, 4 Bengal, 3 Ragdoll, 3 Russian Blue, 2 Japanese Botail, 2 Abyssinian, 2 Exotic Shorthair, 1 American Curl, and 1 Siamese.

The overall Ab-positive rate was 6.7%, ranging from 0% to 10.3% in different cities. (Table 3) The city of Chiayi had highest Ab-positive rate (10.3%, 3/29), followed by Taipei (9.2%, 14/152) and Taichung (8.8%, 6/68). There were four cities (Changhua, *n* = 11 cats; Hsinchu, *n* = 8; Pingtung, *n* = 7; Taitung, *n* = 6) where no Ab-positive cases were reported.

**Table 3:** Prevalence of feline heartworm antigen (Ag) and antibody (Ab) seropositivity in different cities in Taiwan

Area	Sample number	Ag(+)		Ab(+)		Mf(+)	
		(*n*)	(%)	(*n*)	(%)	(*n*)	(%)
Kaohsiung	84	8	9.5%	5	6.0%	2	2.4%
Taipei	152	7	4.6%	14	9.2%	1	0.7%
Taichung	68	2	2.9%	6	8.8%	0	0.0%
Tainan	60	1	1.7%	5	8.3%	0	0.0%
New Taipei	116	1	0.9%	5	4.3%	0	0.0%
Chiayi	29	0	0.0%	3	10.3%	1	3.4%
Yilan	13	0	0.0%	1	7.7%	0	0.0%
Hualien	21	0	0.0%	1	4.8%	0	0.0%
Taoyuan	41	0	0.0%	1	2.4%	0	0.0%
Changhua	11	0	0.0%	0	0.0%	0	0.0%
Hsinchu	8	0	0.0%	0	0.0%	0	0.0%
Pingtung	7	0	0.0%	0	0.0%	0	0.0%
Taitung	6	0	0.0%	0	0.0%	0	0.0%
Total	616	19	3.1%	41	6.7%	4	0.6%

*Ag* Antigen test, *Ab* Antibody test, *Mf* Microfilaria test, *(+)* positive result

The overall Ag-positive rate was 3.1%, ranging from 0% to 9.5% in different cities. (Table 3) The city of Kaohsiung had highest Ag-positive rate (9.5%, 8/84), followed by Taipei (4.6%, 7/152) and Taichung (2.9%, 2/68). There were eight cities (Taoyuan, *n* = 41 cats; Chiayi, *n* = 29; Hualien, *n* = 21; Yilan, *n* = 13; Changhua, n = 11; Hsinchu, n = 8; Pingtung, n = 7; Taitung, n = 6) where no Ag-positive cats were detected.

Among all cats included in this study, 64% (396/616) lived 100% indoors, 13% (79/616) lived >50% indoors, 7% (44/616) lived >50% outdoors and 16% (97/616) lived 100% outdoors. There was no heartworm prevalence difference among each lifestyle group.

Fifteen percent (6/41) of Ab-positive cats were also Ag positive. Sixty-eight percent (13/19) of Ag-positive cats were Ab negative. Microfilariemia was found in four cats, two of which were both Ab and Ag positive while the other two were Ab positive but Ag negative.

For all cats included in this study, 55% (336/616) of presented without clinical signs, 11% (65/616) had cough, 6% (34/616) had dyspnea, 6% (36/616) had GI signs, and 24% (145/616) had other clinical signs. In Ab-positive, but Ag-negative cats, 51% (18/35) had no clinical signs, 11% (4/35) had cough, 6% (2/35) had GI signs, none had dyspnea, and 31% (11/35) had other clinical signs. In Ag-positive cats, 22% (4/19) had no clinical signs, 11% (2/19) had dyspnea, 11% (3/19) had cough, 11% (2/19) had gastrointestinal signs, and 44% (8/19) had other clinical signs.

Multiple logistic regression results for risk factors of feline heartworm Ab seropositivity are shown in Table 4. There was no significant correlation among clinical signs, lifestyle, or Ab seropositivity.

**Table 4:** Results of multiple logistic regression on risk factors for feline heartworm antibody (Ab) seropositivity

		Regression coefficient (B)	Standard Error (SE)	*P* value	Odds Ratio (O)R	95% Confidence Interval (CI)
Lifestyle	100% indoor	reference		0.672		
	> 50% indoor	−0.772	0.628	0.219	0.462	0.135–1.583
	> 50% outdoor	−0.175	0.640	0.784	0.839	0.240–2.940
	100% outdoor	−0.037	0.449	0.934	0.963	0.399–2.324
Dyspnea	No	reference				
	Yes	−0.553	1.046	0.597	0.575	0.074–4.464
GI signs	No	reference				
	Yes	0.058	0.770	0.940	1.059	0.234–4.789
Cough	No	reference				
	Yes	0.602	0.495	0.224	1.825	0.692–4.8181
Other clinical signs	No	reference				
	Yes	0.712	0.380	0.061	2.039	0.968–4.296

## Discussion

### Canine heartworm infection

In the canine study, the overall heartworm prevalence rate (Table 1) in Taiwanese pet dogs was 22.8%, which is slightly lower than a previous report of 26.5% [13]. In Hsinchu and Tainan, the prevalence was slightly decreased, but in other southern and eastern cities (Kaohsiung, Taitung, Yilan, Pingtung), the prevalence rate has increased dramatically. The biggest change was in Hualien. In our study, Hualien had the highest rate of HWI, 51.1%, but in Wu & Fan’s earlier study, Hualien had lowest rate of 4%. [13] In central Taiwan cities (Taichung, Changhua), the prevalence rate of our study was higher than previous reported [16]. In Taipei city, the current prevalence rate was slightly lower than that seen in a previous study [12]. These comparisons suggest that even though the overall prevalence rate was slightly decreased, which may be attributed to the decrease in prevalence in northern Taiwan, canine heartworm has become more prevalent in recent years in eastern, central, and southern Taiwan. Our canine heartworm prevalence rate was similar to earlier studies in adjacent countries, including China, Korea, and Japan [17–19].

Many factors can influence heartworm infection prevalence rates, including animal selection (pet dogs or stray dogs, on prevention or not on prevention), diagnostic test selection (ELISA Ag test, microfilaria test, or PCR), and climate conditions over the previous several years. The present study was designed to match the clinical experience, reflective of what veterinarians actually see, with all subjects being actual hospital patients. This more representative population in our study allows our data to be used by Taiwanese veterinarians as a tool for diagnostic decision making and client education.

An outdoor lifestyle was one of the major risk factors for canine heartworm disease, likely because of increased mosquito exposure in outdoor environments [20]. Our study indicated that, compared with 100% indoor dogs, those spending more than 50% of the time outdoors were 5.5 times more likely to contract HWI; for 100% outdoor dogs, the infection rate was 13.4 times higher. There was no significant difference in heartworm prevalence rate between 100% indoor dogs and dogs spending less than 50% of time outdoors (Table 2). On the other hand, we found that 100% indoor housing did not completely protect the dogs from HWI. There were 43 heartworm-positive dogs living 100% indoors (9.1% of all HWI cases).

The variation in the canine heartworm prevalence rate among lifestyle groups was found in every city studied. We also found that the overall prevalence rate in each city was largely related to the distribution of lifestyle group. Higher outdoor housing frequency result in higher overall prevalence of HWI. The pet dogs’ indoor/outdoor housing frequency difference between cities may due to human population density and housing variability. For example, Taipei, the biggest city in Taiwan, has the highest population density, with most people live in small apartments without yards or balconies. Therefore, most pet dogs live indoors. To the contrary, Hualien has lower population density and most people live in townhouses with yards, so there is space for pet dogs to more readily spend time outside of the house.

In conclusion, the overall prevalence rate in each city appears to be influenced by multiple factors. We suggest that a finding of lower heartworm prevalence does not *necessarily* indicate lower regional risk of potential HWI, as mosquito exposure plays an important role. Therefore, when assessing risk of HWI, lifestyle of pet and owner must also be considered.

We followed American Heartworm Society (AHS) guidelines to diagnose canine heartworm disease with both Ag and microfilaria tests. False-negative Ag test results may occur when the worms are still immature, worm burden is very low, in all-male infections, or with the formation of immune-complexes [21]. Compared with the Ag test, the microfilaria test has lower sensitivity, with 20% of heartworm-infected dogs not being microfilaremic [21]. Microfilaria testing can be performed using direct blood smear, buffy coat smear, millipore filtration, or the modified Knott test [22]. The latter is suggested in the AHS guidelines, but since not all hospitals in this study had the ability or equipment to do the modified Knott test, we chose the direct blood smear method with 0.5 mL blood. In our study, 63% (295/471) of heartworm-positive dogs were both microfilaria and Ag positive, and 35% (166/471) of them were Ag positive but microfilaria negative. False-negative microfilaria test results were more common than described in the AHS guidelines, probably reflecting the methodology used in study. There were 10 Ag-negative cases that had microfilaria in the blood, supporting the AHS Guidelines’ recommendation to perform both microfilaria and Ag tests on every patient.

Clinical signs of heartworm-infected dog are mostly respiratory signs. In mild or early infections, cough alone may be noted. In more severe and chronic cases, dyspnea, exercise intolerance, or right-side heart failure signs may be present [21]. In this study, 34% (159/471) of heartworm-positive dogs were asymptomatic. Cough (28%, 133/471) and dyspnea (13%, 61/471) were the two most frequently seen clinical signs in heartworm-positive dogs. On the other hand, heartworm prevalence rates in dogs presented with cough (*n* = 282) and dyspnea (*n* = 120) were 47% and 51%, respectively. Statistical analysis also indicated that having cough or dyspnea was associated with an increased risk of HWI. When analyzing cough and dyspnea signs in different lifestyle groups, for dogs with cough or dyspnea that lived more than 50% of time outdoors, the HWI prevalence rate was increased to 73%. For dogs with cough or dyspnea spending less than 50% of time outdoors, the HWI prevalence rate was decreased to 15% to24%. These results indicate that unprotected outdoor dogs presenting with cough or dyspnea were at the highest risk for HWI.

### Feline heartworm infection

Feline HWI is very challenging to veterinarians for several reasons. One reason is that in addition to the lung damage caused by adult heartworms, immature worms can cause heartworm-associated respiratory disease (HARD) without a mature infection resulting [23, 24]. Antigen testing is used to detect adult worm Ag. An Ag-positive result confirms the presence of mature female heartworm(s). Antibody testing is used to detect antibodies formed in response to heartworm larvae. An Ab-positive result confirms that the cat is at risk, and that detected antibodies may indicate a mature infection, ongoing exposure to heartworm larvae, or previous exposure [25]. False-negative Ag test results may be due to low worm burden, only male worm infection, immature worms or Ag–Ab complexes [26, 27]. False-negative Ab test results may be due to an inadequate Ab response after exposure or pre-patent infections (<3 month) [15, 28]. A negative Ag or Ab test result cannot completely rule out the HWI or exposure [24, 29].

In the feline study, our Ag prevalence rate in Taipei was higher than previously reported [14], which may indicate an increasing prevalence of feline heartworm disease in the past 11 years. The Ab prevalence rate in Taipei, however, was lower than that found in a previous study [15]. This difference may be due to our study having fewer cats with lower airway clinical signs (18%) than in the previous study (45%). Compared with other Asian reports, our Ag prevalence rate (3.1%) was similar to those in Japan (3%) and Korea (2.6%) [30, 31]. Our Ab prevalence rate (6.7%) was lower than report from the United States (12%) [32]. Our study detected Ab-positive cats in every city except Changhua, Hsinchu, Pingtung, and Taitung. Each of these cites had less than 12 cases (Changhua, *n* = 11; Hsinchu, *n* = 8; Pingtung, *n* = 7; Taitung, *n* = 6), which may be the reason that no infected or Ab-positive cats were detected. Based on the rest of prevalence data in Taiwan, we confirmed that heartworm exposure in pet cats is widely seen in Taiwan and we believe that *all* cats are at risk for heartworm exposure. Year-round heartworm prevention for cats in Taiwan is suggested by our study results, although further and larger studies are needed to fully understand the epidemiology of heartworms in cats in Taiwan.

While most studies have suggested that outdoor exposure was a risk factor for feline heartworm exposure [15, 25, 33], one study found no correlation between outdoor exposure time and heartworm exposure [34]. In the current study, we also did not show correlation between outdoor exposure and heartworm exposure, but, importantly, we found, as did previous studies, that 100% indoor cats were still at risk for heartworm exposure. Heartworm prevention is suggested for all pet cats, regardless of lifestyle.

Fifteen percent (6/41) of our Ab-positive cats were also Ag positive, which indicated they were not only being exposed to heartworm but also had at least one adult female heartworm. All six of these cats were presented with clinical signs (two with cough, one with dyspnea, three with other clinical signs). Sixty-eight (13/19) of our Ag-positive cats were Ab negative. Of these 13 cats, 4 had no clinical signs and 9 had clinical signs (one with cough, one with dyspnea, two with GI signs, five with other clinical signs). The finding of cats that were Ag positive but Ab negative may be due to failure to produce detectable antibodies in infected cats [28], or low sensitivity of the Ab test [29], or simply the small numbers of Ag-positive cats.

Previous studies have shown 10% to 20% of cats with proven HWI to be Ab negative [25]. Some authors advocate using the Ab test as a screening test for feline HWI, then using the Ag test as a confirmation test when a positive Ab test result is found [25, 33]. Because we found a high incidence of false-negative Ab test results in Ag-positive cats, we suggest when screening for HWI in cats, Ag and Ab tests should be done together to get higher diagnostic sensitivity [23, 24, 26].

Clinical signs of feline heartworm disease are more complicated than canine heartworm disease [25]. With each different infection stage, the infected cat may show mild to severe, chronic to acute clinical signs. Respiratory signs are most common. Neurologic and GI signs may also be noted [24]. In this study, 44% of Ab-positive cats presented without clinical signs, 15% had cough, 5% had GI signs, 2% had dyspnea, and 34% had other clinical signs. In Ag-positive cats, 21% had no clinical signs, 16% had cough, 11% had dyspnea, 11% had GI signs, and 42% had other clinical signs. Therefore, only 39% of Ag-positive cats presented with the most common heartworm clinical signs (cough, dyspnea, GI signs), making the diagnosis of heartworm in cats a more challenging task. The presence of cough and dyspnea did not have significant correlation to heartworm exposure in our study, similar to a previous report [15]. There was no significant difference between Ab and Ag positive rates between the group with cough or dyspnea and the group with no cough or dyspnea.

Our study had some limitations. First, case numbers in some cities were not large enough to ensure that they accurately represented regional prevalence. This may have resulted in over- or underestimation of prevalence rates in certain cities, with specific concerns that feline heartworm prevalence underestimation may have occurred in sites where we detected neither Ag- nor Ab-positive cases. Small numbers of cats in some areas make firm conclusions difficult. Second, our study did not have necropsy confirmation. A false-negative or false-positive Ag and Ab result may cause underestimation or overestimation of the heartworm prevalence rate in both dogs and cats. This is more often of concern in cats [29]. Third, because all patients in our study were pet dogs and cats from veterinary hospitals, our study does not answer the question of heartworm prevalence in stray dogs or owned dogs and cats not receiving veterinary care. Fourth, the heat treatment technique was not used on negative Ag samples, which would likely have increased canine results by approximately 7% and feline results by even more.

## Conclusions

The overall prevalence rate of heartworm disease in unprotected pet dogs in Taiwan was 22.8%. Southern and eastern Taiwan had higher prevalence rates than northern Taiwan. Pet dogs that spent more than 50% of their time outdoors that were presented with cough or dyspnea had a higher risk for heartworm disease. We also confirmed that feline heartworm exposure exists in most parts of Taiwan, with an overall Ab prevalence rate of 6.7% and an Ag prevalence rate of 3.1%. Lifestyle had no statistically significant impact on heartworm exposure in pet cats, including the important fact that living indoors did not fully protect pet cats from heartworm exposure. Based on our study result, we suggest that heartworm prevention should be administered to all pet dogs and cats in Taiwan.

## Additional file

Additional file 1: Participating Veterinary Hospitals. (DOCX 13 kb)

## Abbreviations

Ab: Antibody
Ag: Antigen
AHS: American heartworm society
ELISA: Enzyme-linked immunosorbent assay
GI: Gastrointestinal
HARD: Heartworm-associated respiratory disease
HWI: Heartworm infection
