# Supplementary material for: Prevalence and epidemiology of canine and feline heartworm infection in Taiwan
Source: Parasit Vectors. 2017 Nov 9;10(Suppl 2):484. doi: 10.1186/s13071-017-2435-7 (PMC5688419; doi:10.1186/s13071-017-2435-7)
Supplement: Additional file 1: — Participating Veterinary Hospitals. (DOCX 13 kb) [file 13071_2017_2435_MOESM1_ESM.docx]

**Additional file 1**

**Participating Veterinary Hospitals:**

My Friend Animal Hospital

Le Ren Animal Hospital

Shuang Shi Veterinary Hospital

Yung Shin Animal Hospital

Gsiang Animal Hospital

Li Cheng Animal Hospital

Jo Jo Animal Hospital

Rainbow Animal Hospital

Duma Animal Hospital

Jong-Shing Animal Hospital

Stars Animal Hospital

Sannofi Animal Hospital

Hope Animal Hospital

Takahashi Animal Hospital

Sunshine Animal Hospital

Tzu Chi Animal Hospital

Geans Animal Hospital

Johnson Animal Hospital

Hill Top Pet Hospital

Ji Lee Animal Hospital

Chung Hua Animal Clinic

Angel Animal Hospital

Chung Jen Animal Hospital

Loving Kindness Animal Hospital

Liren Animal Hospital

Zhang Hong Animal Hospital

Jen Ji Animal Hospital

Daren Animal Hospital

Ruifang Ai Hsin Animal Hospital

Ho Hsin Animal Clinic

NCYU Animal Hospital

Shulin Ai Hsin Animal Hospital

Southeast Animal Hospital

Chungli Top Vet Animal Hospital

Jia Jia Animal Hospital

Woodpecker Animal Hospital

Bao Chuang Animal Hospital

Blue Sky Animal Hospital

Love of All Vet Hospital

Cardiospecial Veterinary Hospital

Contain animal Hospital

Po-hsin Animal Hospital

Kiwi Animal Hospital

Goodteam Vet Clinic

Yuli Animal Hospital

Maji Vet Hospital

Fei-yaung Animal Hospital

Hung Sheng Animal Hospital

Love Me Animal Hospital

Cheng Yi Animal Hospital

Sin Sin Animal Hospital

Long Shine Animal Hospital

We Care Animal Medical Center

De Sheng Animal Hospital

Leo Fu Animal Hospital

Yo Kong Animal Hospital

Long En Animal Hospital

Luke Animal Hospital

Zion Animal Hospital

Cambridge Animal Hospital,

Ho Me Animal Hospital

Medclover Animal Hospital

Wei Kong Animal Hospital

Chung En Animal Hospital

Cheng Ji Animal Hospital

Life Care Animal Hospital

James & Herriot Animal Hospital

Hsinchu Hsin Ann Animal hospital

Fly Animal Hospital

Fun Young Animal Hospital

Mei Sheng Animal Hospital

Yong Chang Animal Hospital

Rih Kang Veterinary Hospital

Hearts Animal Clinic

Guardian Animal Hospital

Manhattan Veterinary Hospital

Yo Ren Animal Clinic

Maya Animal Hospital

Ri Xiang Animal Hospital

Dr. Paws Animal Hospital

Vet Geek Animal Hospital

Lih Ann Pet Hospital

Eden Animal Hospital

Sam Veterinary Animal Clinic

Dachun Veterinary Hospital

Isee Veterinary Hospital

Well Care Veterinary Hospital

Kaohsiung National Veterinary Hospital

Bowwho Animal Hospital

Stone Animal Hospital

Shang An Animal Hospital

Min Sheng Animal Hospital

Wuquan Animal Hospital

Ren Ren Animal Hospital Bade Branch

Dr. Ma Animal Hospital

Tzuoo Ann Animal Hospital

Green Light Vet Hospital

Ann Shun Animal Hospital

Chun Shan Animal Hospital

Ta Tung Animal Hospital
